# Treatment with endoscopic transnasal resection of hypothalamic pilocytic astrocytomas: a single-center experience

**DOI:** 10.1186/s12893-021-01113-6

**Published:** 2021-02-25

**Authors:** Zhuo-Ya Zhou, Xiao-Shu Wang, Yang Gong, Ode La Ali Musyafar, Jiao-Jiao Yu, Gang Huo, Jia-Min Mou, Gang Yang

**Affiliations:** Department of Neurosurgery, The First Affiliated Hospital of Chongqing Medical University, Chongqing, China

**Keywords:** Endoscopic transnasal resection, Hypothalamic, Pilocytic astrocytomas, In situ bone flap

## Abstract

**Backgrounds:**

Pilocytic astrocytomas (PAs) are World Health Organization (WHO) grade I tumors, which are relatively common, and are benign lesions in children. PAs could originate from the cerebellum, optic pathways, and third ventricular/hypothalamic region. Traditional various transcranial routes are used for hypothalamic PAs (HPAs). However, there are few studies on hypothalamic PAs treated through the endoscopic endonasal approach (EEA). This study reports the preliminary experience of the investigators and results with HPAs via expanded EEAs.

**Methods:**

All patients with HPAs, undergone EEA in our hospital from 2017 to 2019, were retrospectively reviewed. The demographic data, clinical symptoms, complications, skull base reconstruction, prognosis, and endocrinological data were all recorded and analyzed in detail.

**Results:**

Finally, five female patients were enrolled. The average age of patients was 28.6 ± 14.0. All patients had complaints about their menstrual disorder. One patient had severe bilateral visual impairment. Furthermore, only one patient suffered from severe headache due to acute hydrocephalus, although there were four patients with headache or dizziness. Four cases achieved gross-total resection, and one patient achieved subtotal resection. Furthermore, there was visual improvement in one patient (case 5), and postoperative worsening of vision in one patient (case 4). However, only one patient had postoperative intracranial infection. None of the patients experienced a postoperative CSF leak, and in situ bone flap (ISBF) techniques were used for two cases for skull base repair. In particular, ISBF combined with free middle turbinate mucosal flap was used for case 5. After three years of follow-up, three patients are still alive, two patients had no neurological or visual symptoms, or tumor recurrence, and one patient had severe hypothalamic dysfunction. Unfortunately, one patient died of severe postoperative hypothalamus reaction, which presented with coma, high fever, diabetes insipidus, hypernatremia and intracranial infection. The other patient died of recurrent severe pancreatitis at one year after the operation.

**Conclusion:**

Although the data is still very limited and preliminary, EEA provides a direct approach to HPAs with acceptable prognosis in terms of tumor resection, endocrinological and visual outcomes. ISBF technique is safe and reliable for skull base reconstruction.

## Introduction

Pilocytic astrocytomas (PAs) are relatively benign and slow-growing tumors that occur in children [1]. These lesions in children are almost well-differentiated, low-grade gliomas with a good prognosis [2–4]. However, in patients equal or older than 14 years old, PAs are rare, and have shorter overall survival, when compared with children [5–7]. PAs can originate from any site in the CNS, although the most frequent origin is the cerebellum (42%), followed by the supratentorial compartment (36%), the optic pathway and hypothalamus (9%), the brainstem (9%), and the spinal cord (2%) [8]. The clinical symptoms of PAs vary depending on the tumor size, location and extension, and age at diagnosis. In addition, the most common symptoms and presenting signs are headaches, visual disturbances and hypothalamic dysfunction [3, 9].

Taking the tumors critical location, the histologically benign nature, and the considerable preoperative or postoperative risk all into consideration, PAs are very difficult to manage. These tumors are usually approached and resected through the anterior or anterolateral transcranial route [10, 11]. Alternatively, a few recent small series have shown that endoscopic transnasal resection is safe and feasible for hypothalamic PAs (HPAs) [10, 12]. Over the past years, there have been significant advances and development for the EEA, which has expanded the limitation of pituitary tumors. This approach provides a clear and direct visualization to the lesion areas. Thus, the neurosurgeons could avoid any brain manipulation or retraction, and achieve better manipulation. The previous study showed that extended EEA might be a promising approach for selected cases with parasellar, sellar or clivus lesions [13, 14]. In addition, Bin Abdulqader et al. represented their experience of removing the pediatric optic pathway gliomas through EEA with acceptable perioperative outcomes [15]. However, EEA has rarely been applied to remove HPAs. Furthermore, the reconstruction of the skull base during the EEA is very important for the prognosis of patients. The investigators attempted to apply the novel ISBF to the repair procedure, and obtained satisfactory results for the expanded EEA approach [16–18]. In the present study, the investigators attempted to share their experience, and the results for the EEA conducted for five patients with HPAs, and initially evaluates the safety and effectiveness of the ISBF, when applied to patients with HPAs.

## Methods

### Patients

The investigators retrospectively reviewed all patients with HPAs treated using endoscopic transnasal resection in our hospital from 2017 to 2019. And all adult patients gave written informed consent to their participation in the study, which followed the ethical guideline of the Declaration of Helsinki. As for the patients younger than 18 years old, her parents had written informed consent to her participation in this study. Each procedure was performed by the same neurosurgeon who had rich experience of EEA. The demographic data, clinical presentations, preoperative and postoperative complications, clinical prognosis, endocrinological, and ophthalmological were all recorded and analyzed in detail. In addition, the compromise of hypothalamic function was carefully analyzed, including the assessment of the body mass index (BMI), compulsive hyperphagia, and psychic or behavioral alterations. Indeed, magnetic resonance imaging (MRI) and computed tomography angiogram (CTA) were conducted for every patient, preoperatively, and these were initially repeated 1 month after surgery. The, these were repeated after 3 months and 6 months, and annually thereafter.

### Endoscopic endonasal approach

**Fig. 1:**
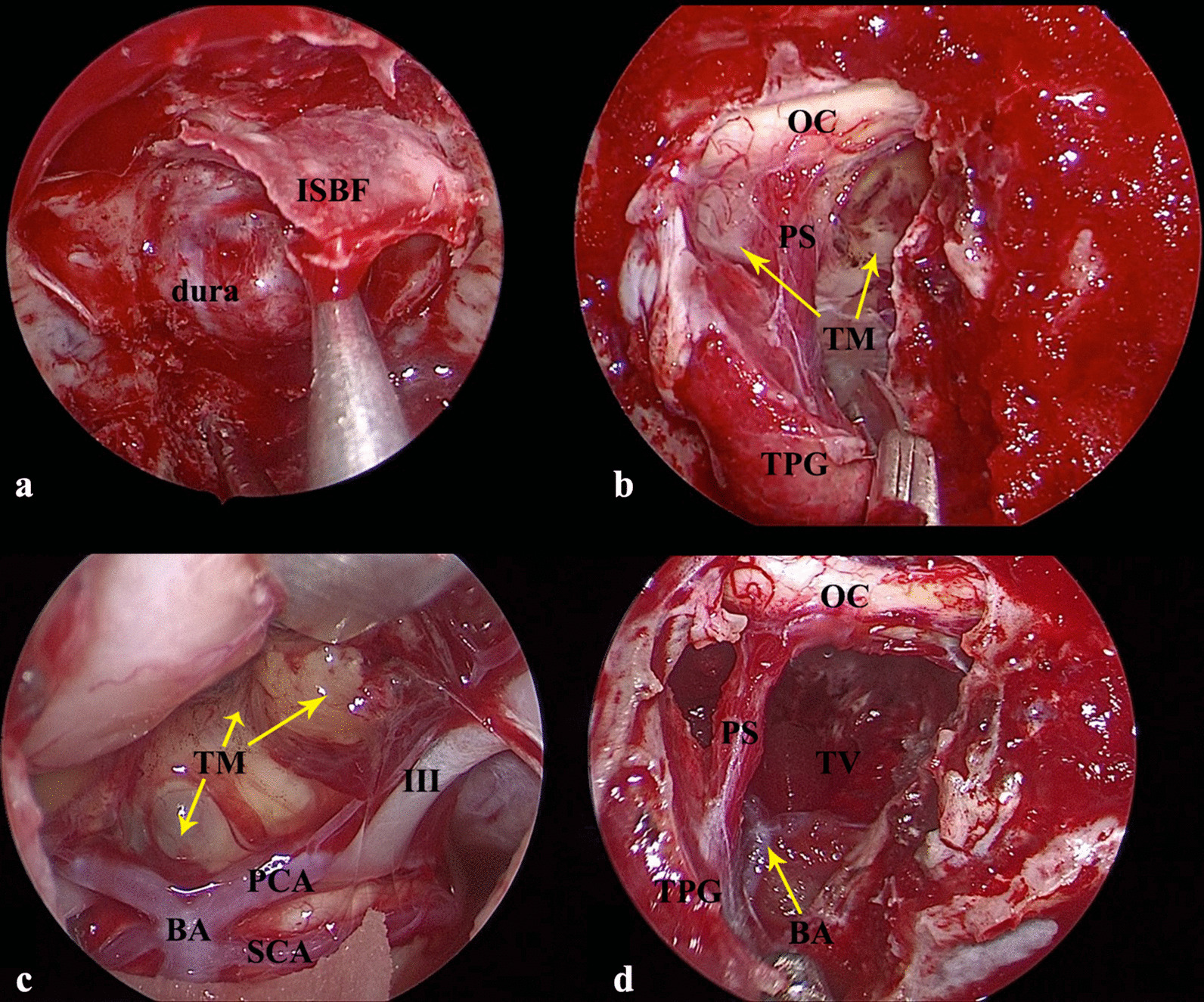
The intraoperative view, a 0° angled scope. **a** Elevation of the In Situ Bone Flap from the dura below it using a Cottle dissector; B: Endoscopic view showing the tumor after pituitary transposition; *PS* pituitary stalk, *PG* pituitary gland, *OC* optic chiasm, *TM* tumor. **c** Endoscopic view showing the tumor; *III* oculomotor nerve; *BA* basilar artery, *PCA* posterior cerebral artery, *SCA* superior cerebellum artery, *TM* tumor. **d** After the tumor removal, the endoscopic view shows the exposure of the third ventricle (TV) with the resulting high-flow CSF leakage, and the pituitary stalk (PS), optic chiasm (OC) and transposed pituitary gland (TPG)

Previous studies have described the EEA in detail [19, 20]. Briefly, a two nostrial approach was used for all five patients. Under general anesthesia via an endotracheal tube, the patient was positioned in the supine position, with the thorax slightly elevated on the operating table. The approach started with a right side of the middle turbinate resection and antrostomy, while the contralateral one was laterally displaced. The nasal cavity was sufficiently expanded. Prior to the posterior septectomy, a vascularized pedicle nasoseptal flap was harvested from the right nasal cavity, according to the method introduced by Hadad [21] and Kassam [22], except for case 5. Once harvested, the flap was stored into the nasopharynx [21]. Once the wide sphenoidotomies and posterior ethmoidectomies were performed, the bones of the tuberculum sellae and sella were drilled with a 4-mm diamond burr for three patients, and the “In Situ Bone Flap” (ISBF) was performed for the other two patients (case 3; Fig. 1; case 5; Fig. 3a–c) with a 2.5-mm diamond burr. The techniques for ISBF have been described in detail elsewhere [16–18]. The suprachiasmatic translamina terminalis corridor was used to access the tumor of case 2 and 4. With a technique similar to microsurgical resection, the resection started with central debulking using suction and an ultrasonic aspirator. The tumor feeding vessels were cauterized and cut, and the tumor was bimanually carefully dissected from the surrounding structures. It is important to preserve the superior hypophyseal artery and its tiny feeding branches to the optic chiasm. After the extirpative phase of the surgery was concluded, the skull base defect was routinely reconstructed with the pedicled nasoseptal flap (PNSF), including intradural placement of the dura substitute (Heal-All, ZH-BIO, Inc., Yan-Tai, China) and extradural placement of an autologous fascia lata graft harvested from the thigh and the PNSF. An ISBF was used for case 3 and 5. The NasoPore (Ethicon, Inc., USA) and iodoform gauze were inserted to buttress the repair. Intraoperative lumbar drainage was not routinely used for all five cases.

## Results

Finally, five female patients were enrolled into the present study. As shown in Table 1, the mean age at surgical resection was 28.6 ± 14.0 years old (range: 14–46 years old). The mean size of the lesions was 35.0 ± 9.0 mm (range: 25–46 mm). The investigators did not identify any patients with positive NF1. After careful evaluation with visual box, we only found one patient with severed bilateral visual impairment, while three patients had compression of the optic chiasma. The other symptoms were headache or dizziness (n = 4, 80%), amenhorrea or menstrual disorder (n = 5,100%), weight gain (n = 1, 20%), and memory deterioration (n = 1, 20%). According to the preoperative endocrine examinations, there were only two patients with abnormal preoperative pituitary function, which included hyperprolactinemia (case 2) and low free thyroxine (case 3). The demographic and clinical characteristics were summarized detailly in Table 1.

**Table 1:** Patient’s demographic and clinical characteristics

	Case 1	Case 2	Case 3	Case 4	Case 5
Age	46	20	41	14	22
Gender	Female	Female	Female	Female	Female
Clinical presentation	Bilaterla visual disturbance, headache, amenhorrea	Headache, amenhorrea, weight gain	Amenhorrea,dizziness memory deterioration	Menstrual disorder, headache	Menstrual disorder
Preoperative pituitary function	Normal	Hyperprolactinemia	Low free thyroxine	Normal	Normal
Tumor size (mm)	43 × 42	30 × 25	31 × 30	46 × 45	25 × 21
Intratumoral cyst/calcification	Solid and Cyst	Cyst	Solid and Cyst	Solid and Cyst	Solid and Cyst
Displacement of chiasm	Yes	Yes	No	Yes	Yes
Resection	Gross-total	Gross-total	Gross-total	Subtotal	Gross-total
Preservation of stalk	No	Yes	Yes	No	Yes
Histologic type	PA	PA	PA	PA	PA
Postoperative pituitary function	Hypopituitarism	Hyperprolactinemia	Hypopituitarism	Hypopituitarism	Hypopituitarism
Vision outcomes	Die	Improved	No change	Deterioration	No change

**Fig. 2:**
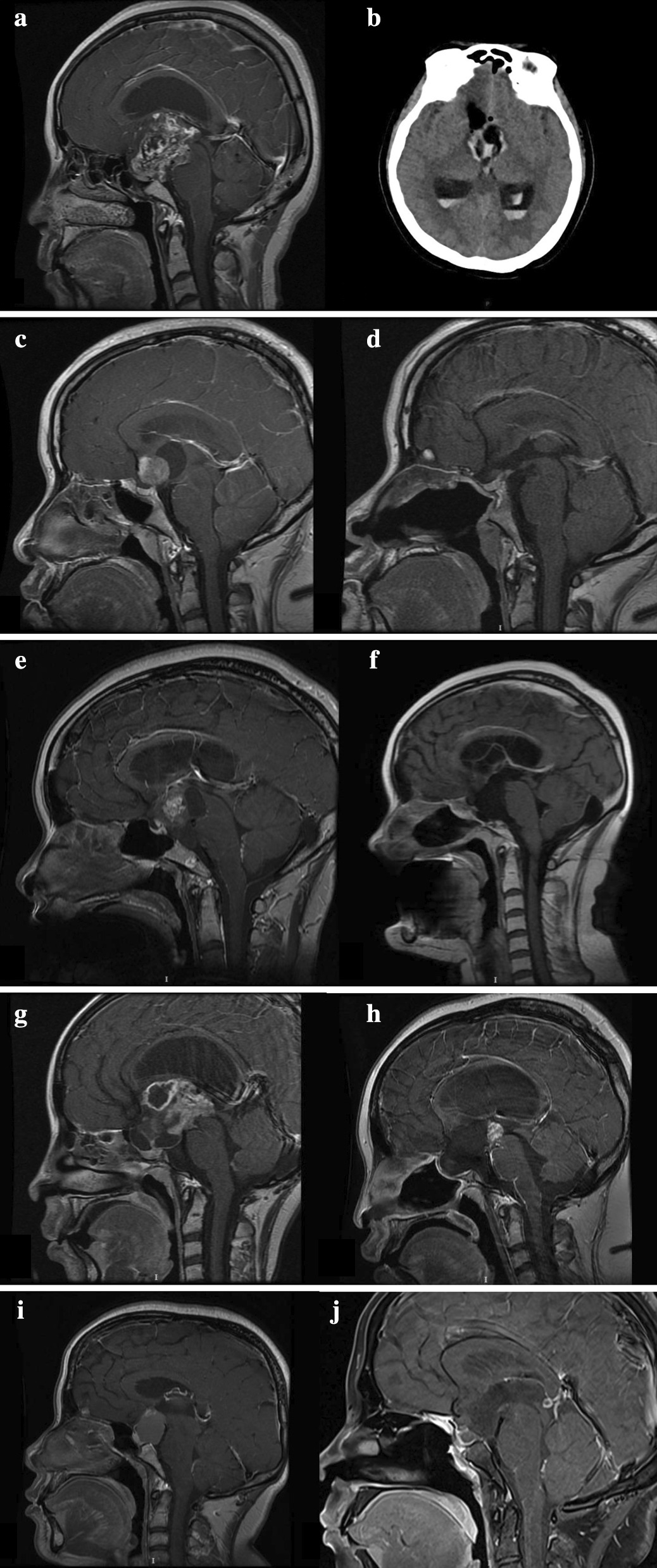
Comparison of the sagittal MRI obtained at pre- and post-operation. **a**, **b**: Case 1. The preoperative MRI shows a giant hypothalamic tumor with no clear margin between the tumor and hypothalamic structure. The postoperative CT (after one day) shows the GTR of the tumor and hematocele in the lateral ventricles. **c**, **d**: Case 2. The preoperative MRI study shows the suprasellar tumor with solid and cystic portions. The postoperative MRI study obtained at one year after surgery shows that the gland and the stalk were preserved and visible. The tumor was radically resected. **e**, **f**: Case 3. The preoperative MRI shows the tumor located suprasellar, interpeduncular and prepontine cistern. The postoperative MRI (6 months) shows the radical resection of the hypothalamic glioma. **g**, **h**: Case 4. The preoperative and postoperative (one year) MRI shows the suprasellar hypothalamus glioma with the involvement of the third ventricle. The residual tumor remained stable after the subtotal resection. **i**, **j**: Case 5. The preoperative and postoperative (6 months) MRI shows the radical resection of the hypothalamic glioma

All five patients did not undergo any preoperative treatment. The preoperative MRI studies were shown in Fig. 2. Four of these patients underwent gross-total resection through EEA, while the remaining patient underwent subtotal resection. All five patients were without any intraoperative complications. Furthermore, all five patients underwent skull base reconstructive procedure with PNSF. Moreover, ISBF combined with PNSF was used for the reconstruction in case 3, and ISBF combined with free middle turbinate mucosal flap was used for case 5. The postoperative histopathological diagnosis determined pilocytic astrocytoma with WHO grade I for all cases. One patient had preoperative hyperprolactinemia, which remained unchanged, postoperatively. Furthermore, four patients presented with postoperative hypopituitarism. All five patients suffered from temporary diabetes insipidus postoperatively with rapid recovery one week after surgery. And one patient (case 4) underwent extra ventricular drainage for acute hydrocephalus, preoperatively. One patient had intracranial infection postoperatively. Unfortunately, this patient even died due to a severe postoperative hypothalamus reaction at 1 month after surgery (case 1). Besides, there was only one patient (case 4) with visual deterioration after evaluation through visual box postoperatively. No recurrence was found for case 2 and 5, and the residual tumor was stable in case 4. All patients were not treated with radiotherapy or chemotherapy.

## Discussion

PAs are the most benign and slow-growing tumors that occur in children. However, these tumors might be malignant and contribute to shorter survival time in patients not younger than 14, when compared to children [5–7]. In addition, approximately 9% are PAs with hypothalamus [8]. Furthermore, there are few studies on HPAs, and the reason is mainly because of its low incidence. The present study summarized the treatment and prognosis of these tumors in our hospital.

According to the origin of PAs, the investigators were able to classify these tumors into lesions that arise in the anterior visual pathway and posterior visual pathway, including the optic chiasm, hypothalamus, and third ventricle [23]. In addition, patients with tumors that originated from the posterior visual pathway were more likely to complain of bilateral loss of visual field and visual acuity [24]. Patients with NF1 are more likely to undergo long-term observation and conservative management, because these tumors might be asymptomatic, and remain as a stable disease condition [11]. For symptomatic young patients, chemotherapy is the first-line treatment strategy [11, 25, 26]. However, the investigators did not find any patient with positive NF1.

Due to its deep location and the presence of critical neurovascular structures, the histologically benign nature, and the considerable risk of complications following surgical intervention, there are a number of controversies on the management of HPAs. HPAs might have the potential to rapidly progress, remain stable, or spontaneously regress [27]. Primary surgical resection is not a standard treatment. The goals of surgery were to establish the diagnosis, debulk the tumor mass, and improve the mass effect-related symptoms. Neurosurgeons can perform surgical resection for patients with large exophytic or cystic tumors [28]. The balance of the extent of the tumor resection, visual compromise and preservation of hypothalamic function should be prudently evaluated before surgery. Initially, for the first patient (case1), preoperatively, the diagnosis was suspected to be a craniopharyngioma. This suspicion arose due to some misleading neuroradiological features at the neuroimaging. The preoperative MRI study revealed an irregular mass in the suprasellar area. The boundary of the mass was unclear, and the signal was uneven. On the edge of the lesion, there was a strip low signal shadow. On the contrast-enhanced scan, the lesions had an inhomogeneous enhancement, while some had a ring-shaped enhancement, but there was no obvious enhancement found in some internal areas (Fig. 2a). Intraoperatively, the lesion was found to be located in the suprasellar region, extending into the third ventricle posteriorly and upward. Furthermore, intraoperatively, it was found that the tumor was solid, with an abundant blood supply and uneven texture (some of which were thin and soft, while some were tough). There was no obvious capsule boundary between the tumor and the wall of the third ventricle, and the intraoperative histopathological diagnosis was chronic gliosis. At the end, the tumor was radically removed, and the floor of the third ventricle was recognizable and free from euplastic remnants. However, the patient died due to a severe postoperative hypothalamus reaction that presented with coma, high fever, diabetes insipidus, hypernatremia and intracranial infection. Furthermore, the aggressive surgery and excessive heat from the bipolar coagulation might have caused the damage of the hypothalamus. As for case 4, the investigators chose a subtotal resection, in terms of the tumor’s extent of adhering to the surrounding tissues, and the preservation of hypothalamic function. After surgery, this patient had visual deterioration. The visual deterioration might be caused by the trans-lamina terminalis approach. Silva et al. reported postoperative visual deterioration in 4 cases among 29 patients with trans-lamina terminalis approach [29]. Conversely, we found the other patient (case 2) with trans-lamina terminalis approach even had mildly visual improvement in our study. Thus, we draw the opinion that trans-lamina terminalis approach might have partial effect on the vision. However, more study should be conducted to confirm this concept. Except for the visual defects, this patient reached a good recovery, although the patient continued to have severe hypothalamic dysfunction. The postoperative images revealed that the residual tumor remained stable after subtotal resection (Fig. 2h). To our knowledge, gross-total resection of PAs is of prognostic importance, although this has a high risk of injury to the optic apparatus, hypothalamic-pituitary axis, and the carotids [30–32]. In the present study, four patients underwent gross-total resection through EEA, while the other patient (case 4) underwent subtotal resection. One patient (case 1) died due to a severe postoperative hypothalamus reaction, and one patient (case 4) with severe hypothalamic dysfunction survived. These patients still had no visual deterioration or recurrence of HPAs until the present. It is vital to preserve the hypothalamic function and visual, in order to improve the patient quality of life.

Traditionally, tumors that originated from the hypothalamus area were treated with different kinds of transcranial approaches, including pterional, subfrontal, transcortical and transcallosal, or via other minimally invasive approaches, such as the supraorbital eyebrow craniotomy [33–39]. The endoscopic transsphenoidal route could easily access the hypothalamic region without any brain retraction, parenchymal incision and vascular manipulation [40]. These results and the extensive experience of the investigators with suprasellar tumors suggests that the endoscopic transsphenoidal route can also be considered for selected patients with hypothalamic gliomas. EEA has been proven to be an important treatment option for HPAs in few institutions. However, there were few studies on the application of EEA for the treatment of HPAs, especially in patients not younger than 14 [10].

**Fig. 3:**
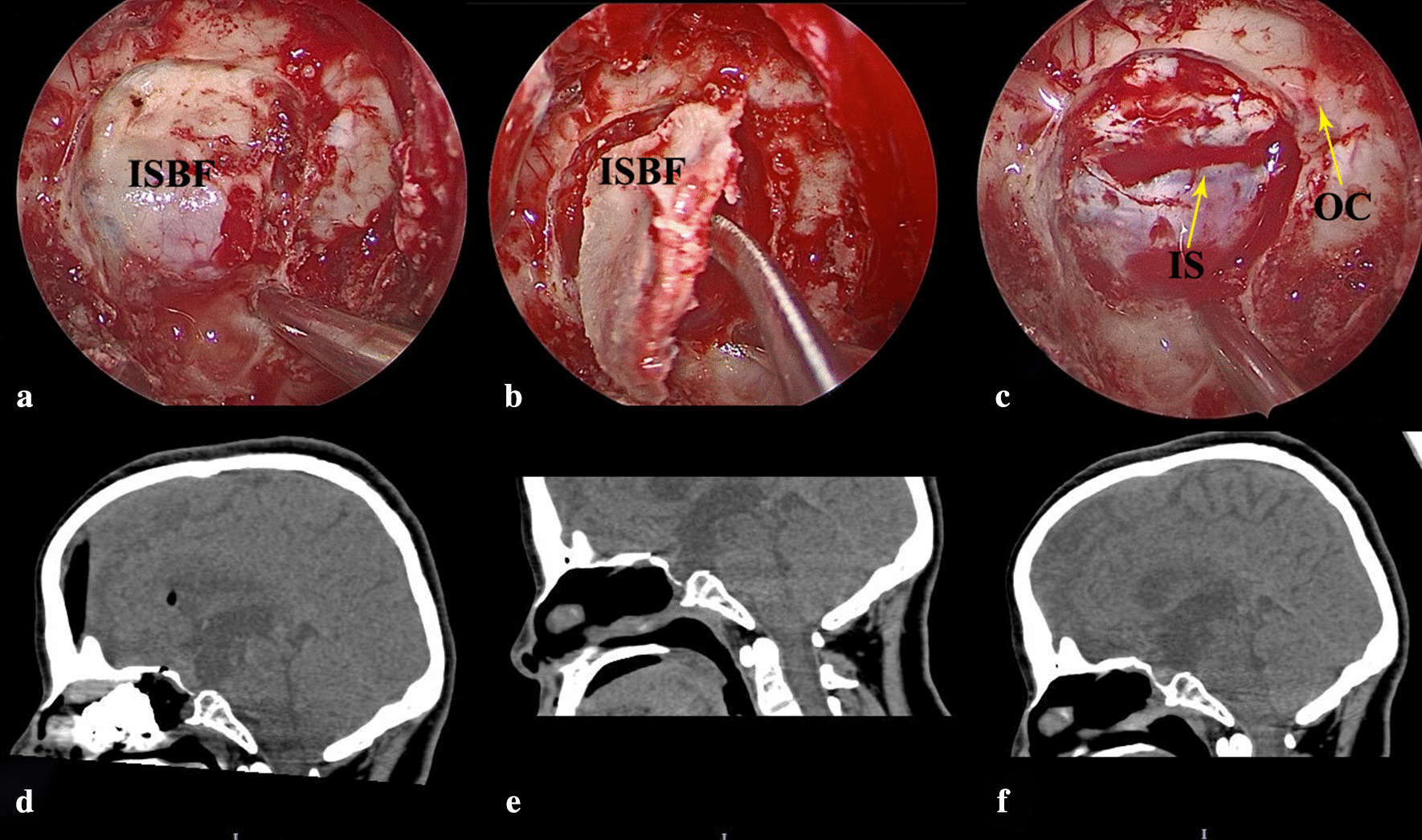
The intraoperative photographs show the harvesting of the “In Situ Bone Flap” and the evolution of the skull base defect postoperative healing. **a** The dimension of the designed ISBF ranged from one lateral cavernous sinus to the other, and from the planum sphenoidale to the sellar floor; **b** The elevation of the ISBF from the dura below it using a Cottle dissector; **c** The endoscopic view shows the intact dura, intercavernous sinus (IS) and optic canal (OC) after removing the In Situ Bone Flap. **d**–**f** The postoperative sagittal CT images shows the process of healing of the In Situ Bone Flap, at one day (**d**), 3 months (**e**) and 6 months (**f**), postoperatively

The reconstruction of the skull base is very important to the prognosis and recovery, postoperatively. In the present study, the traditional reconstruction with PNSF was performed, and the novel procedure with ISBF was successfully performed. The objectives of the skull base reconstruction after EEA are identical to those after traditional transcranial procedures, including the repair of the tissue barrier between the contaminated nasopharyngeal space and sterile intracranial, the establishment of the watertight closure, the elimination of the dead space, the adequate support for the intracranial components, and the reconstruction of the initial anatomical structure [41–43]. According to these goals, it is vital to achieve a rigid reconstruction of the bone defect from the EEA. Unfortunately, conventional reconstructive methods that use the PNSF could not achieve the real reestablishment of these bone defects. Hence, these permanent skull base defects might contribute to the acute or chronic headache, nonspecific discomfort, and pseudo meningocele [17]. However, most of present neurosurgeons argue that the multilayer reconstruction combined with PNSF is enough. Thus, there is no need to reconstruct the bone defects [44]. On the contrary, there are still several rigid reconstruction methods combined with different kinds of materials, including the cartilage, bone, absorbable or nonabsorbable plates, titanium mesh and gasket seal watertight closure technique, and these could achieve the promised postoperative CSF leakage, when compared with traditional techniques [43, 45–47]. However, these materials might lead to injury of the neurovascular structures, compression on the optic nerves, and severe encephalic infection [17, 45]. Thus, the most suitable materials applied to rigid reconstruction might be the patient’s autologous bone, with septal bone grafts collected from the nasal septum, which is the most common [43, 46]. Nevertheless, it is difficult to harvest a sufficient and intact septal bone from the nasal septum to reestablish the bone defect, postoperatively. Considering the conditions above, the investigators attempted to find a more ideal material that could lead to its improvement. The previous study of the investigators concluded that the novel reconstruction combined with ISBF could sufficiently reduce the incidence rate of postoperative CSF leakage [17]. Furthermore, since the ISBF originates from its original location, the ISBF has innate advantages of the convenience of harvesting procedure, excellent biological compatibility with the bone defect, proximity to the original anatomical structures, stable fixation, and rapid bone healing. In the present study, two patients underwent the novel technique combined with ISBF, and these was no postoperative CSF leakage, chronic headache, or nonspecific discomfort. For case 5, ISBF combined with free middle turbinate mucosa flap was used to repair the skull base defect without adding operative time for PNSF harvesting, without requiring external incisions to harvest fat and the fascia, and most importantly, without requiring PNSF with its well-recognized postoperative rhinological morbidity. In the subsequent clinical research of the investigators, it was found that the application of ISBF combined with the free middle turbinate mucosa flap in skull base reconstruction of the expanded EEA obtained an excellent result [16]. In addition, the ISBF achieved stable and rapid bone healing, according to the examination during the follow-up period (Fig. 3, Postoperative sagittal CT images).

However, there were still some disadvantages and potential limitations, which were main due to the small sample size, and absence of randomized comparisons. Unfortunately, there was lack of data on removing HPAs through craniotomy, mainly due to the difficulty of exposure as well as the great risk of craniotomy, thus most patients gave up the treatment via traditional approaches. In addition, the present study was a retrospective study conducted in a single institution. Therefore, it is necessary to conduct further researches to confirm the safety and effectiveness of the EEA, and the reconstruction combined with ISBF among HPAs.

## Conclusion

The preliminary experience of the investigators suggest that the expanded EEA can achieve the gross-total dissection and resection of HPAs, while avoiding any brain manipulation or retraction. EEA is a safe, direct and straightforward approach to HPAs. The balance of the extent of the tumor resection, visual compromise and the preservation of hypothalamic function should be prudently evaluated before surgery. The application of ISBF can reestablish the skull base structure more accurately to its original anatomy, and this was mainly due to the bone defect well-healing. Thus, the investigators were able to achieve a more stable and durable reconstruction, which is equal to the conventional transcranial reconstruction. Briefly, the ISBF closure combined with conventional PNSF after the resection of HPAs via EEA might decrease the incidence of postoperative CSF leakage. Furthermore, the reconstruction by ISBF combined with a free middle turbinate mucosal flap provides a new option. Importantly, further studies about EEA and ISBF are needed to confirm the safety and efficacy, when applied to treat HPAs.

## Data Availability

The datasets used and/or analyzed during the current study are available from the corresponding author on reasonable request.

## Abbreviations

PA: Pilocytic astrocytomas
HPA: Hypothalamic pilocytic astrocytoma
EEA: Endoscopic endonasal approach
ISBF: In situ bone flap
